# Whole-Genome Sequence of *Streptomyces kaniharaensis* Shomura and Niida SF-557

**DOI:** 10.1128/MRA.01434-19

**Published:** 2020-04-02

**Authors:** Wen Zhu, Xuan Liu, Margaret Hughes, Valérie de Crécy-Lagard, Nigel G. J. Richards

**Affiliations:** a School of Chemistry, Cardiff University, Cardiff, United Kingdom; b Centre for Genomic Research, Institute of Integrative Biology, University of Liverpool, Liverpool, United Kingdom; c Department of Microbiology, University of Florida, Gainesville, Florida, USA; d Foundation for Applied Molecular Evolution, Alachua, Florida, USA; University of Maryland School of Medicine

## Abstract

Streptomyces kaniharaensis is a Gram-positive bacterium that produces formycin A 5′-phosphate, a C nucleotide with antimicrobial and anticancer activity. Here, we report the sequencing, assembly, and annotation of the draft genome sequence of *Streptomyces kaniharaensis* Shomura and Niida.

## ANNOUNCEMENT

Streptomyces kaniharaensis, isolated from soil, produces formycin A 5′-phosphate, a C nucleotide exhibiting antimicrobial activity (1). The metabolic pathway to formycin A 5′-phosphate is interesting because little is known about C nucleotide biosynthesis (2). Here, we present the draft genome sequence of *Streptomyces kaniharaensis* Shomura and Niida.

*Streptomyces kaniharaensis* Shomura and Niida (ATCC 21070) was purchased from ATCC (Middlesex, UK). Freeze-dried cells were dissolved in autoclaved tryptic soy broth (TSB) medium containing 25% glycerol. The culture (10 μl) was plated onto TSB agar and incubated at 26°C for 15 days. After this time, one 0.5-inch-diameter mycelium was used to inoculate TSB liquid medium (50 ml). Incubation at 26°C for 5 more days and centrifugation (3,500 rpm) at 4°C gave cell pellets that were washed (2×) with deionized water and stored at −80°C. Genomic DNA extraction was performed using a PureLink genomic DNA minikit (Thermo Fisher, UK) following the protocol for Gram-positive bacteria. Repeating this procedure gave 17 μg of genomic DNA (*A*_260_/*A*_280_, 1.9; *A*_260_/*A*_230_, 2.0), which was not sheared because the size range was suitable for library preparation. Genomic DNA (5 μg) was size selected (6 to 50 kb) on a 0.75% cassette using the Sage Blue Pippin system, purified using AMPure beads (1:1 vol/vol), and checked for concentration (Qubit), and the size distribution was determined (Fragment Analyzer, Agilent). A PacBio DNA library was generated, which was sequenced on an RSII single-molecule real-time (SMRT) cell. PacBio reads were quality controlled on the basis of (i) productivity or yield of the cell, (ii) loading of zero-mode wave guides (ZMWs) based on a Poisson distribution, (iii) the length and quality of polymerase reads, and (iv) ensurance that the reads of each insert were equal to its expected size. PacBio reads were assembled using the Hierarchical Genome Assembly Process (HGAP) pipeline as embedded in the PacBio SMRT link software. Default parameters were used except where otherwise noted. Sequencing generated 13,396 preassembled reads with a mean read length of 7,063 bp. Raw reads were trimmed and filtered for high-quality bases. In total, 130,203 reads were aligned and mapped, leading to 1,470,565,355 mapped subread bases. A draft genome sequence of *Streptomyces kaniharaensis* was assembled from 19 polished contigs with an *N*_50_ contig length of 6,153,183 bp. The size of the assembled genome is 8,774,359 bp, and the G+C content is 72.15%. Annotation was carried out using the NCBI Prokaryotic Genome Annotation Pipeline (PGAP) (3), which identified 8,035 genes, of which 7,532 coding DNA sequences (CDSs) were associated with proteins.

Putative biosynthetic gene clusters (BGCs) were identified using PATRIC database tools (https://www.patricbrc.org/) (4) by searching the proteome (PATRIC identification number 212423.6) for ForH homologs (PDB number 6NKO) (5). The top hit was annotated as IMP cyclohydrolase (EC 3.5.4.10) (locus tag F7Q99_03110), and analyses of proteins encoded by neighboring genes (locus tags F7Q99_03100 to F7Q99_03210) allowed us to identify the BGC for formycin A 5′-phosphate. Our assignment was later verified independently (6). Some enzymes in the BGC have been recently characterized, including ForT, which couples 4-amino-1H-pyrazole-3,5-dicarboxylic acid and phosphoribosyl pyrophosphate (PRPP) (7, 8), and ForI, a pyridoxal 5-phosphate (PLP)-dependent aminotransferase involved in pyrazole synthesis (9).

### Data availability.

The draft genome sequence of *Streptomyces kaniharaensis* Shomura and Niida (BioProject number PRJNA574488) has been deposited in GenBank under the accession number WBOF00000000. The raw reads (SRA numbers SRX7765393 and SRX77655394) are also available. DNA sequences for enzymes in the formycin A 5′-phosphate BGC can be found under the accession number NZ_WBOF01000001 and spanned from RefSeq locus tag F7Q99_03050 to F7Q99_03210 (PATRIC fig|212423.6.regulatory.2 to fig|212423.6.peg.674).

## References

[1] Suhadolnik RJ 1970 Nucleoside antibiotics. John Wiley & Sons, New York, NY.

[2] Ochi K, Yashima S, Eguchi Y, Matsushita K 1979 Biosynthesis of formycin. Incorporation and distribution of ^13^C-, ^14^C-, and ^15^N-labeled compounds into formycin. J Biol Chem 254:8819–8824. 479162

[3] Tatusova T, DiCuccio M, Badretdin A, Chetvernin V, Nawrocki EP, Zaslavsky L, Lomsadze A, Pruitt KD, Borodovsky M, Ostell J 2016 NCBI Prokaryotic Genome Annotation Pipeline. Nucleic Acids Res 44:6614–6624. doi:10.1093/nar/gkw569. 27342282 PMC5001611

[4] Wattam AR, Brettin T, Davis JJ, Gerdes S, Kenyon R, Machi D, Mao C, Olson R, Overbeek R, Pusch GD, Shukla MP, Stevens R, Vonstein V, Warren A, Xia F, Yoo H 2018 Assembly, annotation, and comparative genomics in PATRIC, the all bacterial bioinformatics resource center. Methods Mol Biol 1704:79–101. doi:10.1007/978-1-4939-7463-4_4. 29277864

[5] Wang S-A, Ko Y, Zeng J, Geng Y, Ren D, Ogasawara Y, Irani S, Zhang Y, Liu H-W 2019 Identification of the formycin A biosynthetic gene cluster from *Streptomyces kaniharaensis* illustrates the interplay between biological pyrazolopyrimidine formation and *de novo* purine biosynthesis. J Am Chem Soc 141:6127–6131. doi:10.1021/jacs.9b00241. 30942582 PMC6612245

[6] Ko Y, Wang S-A, Ogasawara Y, Ruszczycky M, Liu H-W 2017 Identification and characterization of enzymes catalyzing pyrazolopyrimidine formation in the biosynthesis of formycin A. Org Lett 19:1426–1429. doi:10.1021/acs.orglett.7b00355. 28233490 PMC5467530

[7] Ren D, Wang S-A, Ko Y, Geng Y, Ogasawara Y, Liu H-W 2019 Identification of the *C*-glycoside synthases during biosynthesis of the pyrazole-*C*-nucleosides formycin and pyrazofurin. Angew Chem Int Ed Engl 58:16512–16516. doi:10.1002/anie.201910356. 31518483 PMC6911263

[8] Zhang M, Zhang P, Xu G, Zhou W, Gao Y, Gong R, Cai Y-S, Cong H, Deng Z, Price NPJ, Mao X, Chen W 2019 Comparative investigation into formycin A and pyrazofurin A biosynthesis reveals branch pathways for the construction of *C*-nucleoside scaffolds. Appl Environ Microbiol 86:e01971-19. doi:10.1128/AEM.01971-19. PMC6952237 31676476

[9] Gao S, Liu H, de Crécy-Lagard V, Zhu W, Richards NGJ, Naismith JH 2019 PMP-diketopiperazine adducts form at the active site of a PLP dependent enzyme involved in formycin biosynthesis. Chem Commun (Camb) 55:14502–14505. doi:10.1039/c9cc06975e. 31730149 PMC6927412

